# Supplementary material for: FraC nanopores with adjustable diameter identify the mass of opposite-charge peptides with 44 dalton resolution
Source: Nat Commun. 2019 Feb 19;10:835. doi: 10.1038/s41467-019-08761-6 (PMC6381162; doi:10.1038/s41467-019-08761-6)
Supplement: Supplementary file 1 — Supplementary Information [file 41467_2019_8761_MOESM1_ESM.pdf]

Supplementary Information

FraC Nanopores with Adjustable Diameter Identify the Mass of Opposite-Charge Peptides with 44 Dalton Resolution

Huang et al.

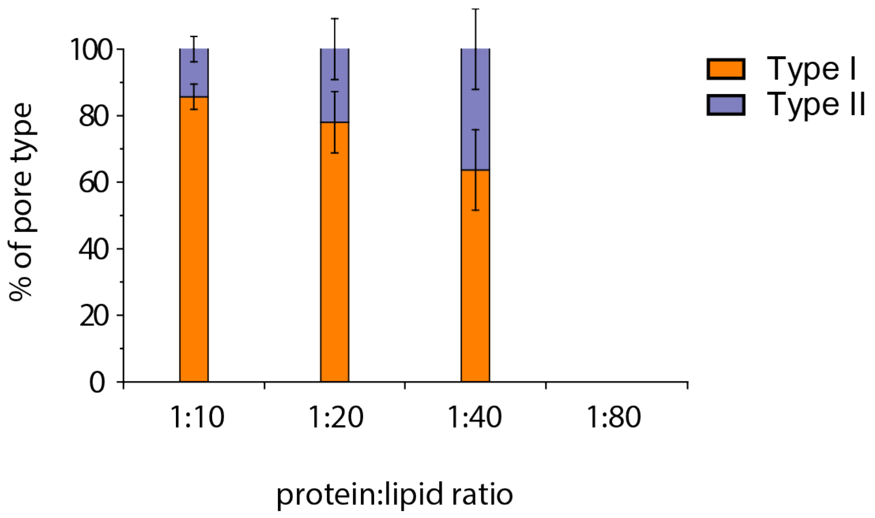

Supplementary Fig 1. Oligomerization at different lipid:protein dilutions. Oligomers were obtained by mixing purified Wt-FraC monomeric proteins (1 mg/mL, 0.5 mg/mL, 0.25 mg/mL, 0.125 mg/mL) with an equimolar volume of lipids (10 mg/mL), thus in a 1:10 ratio, 1:20 ratio, 1:40 ratio and 1:80 ratio to increase the fraction of type II Wt-FraC nanopores that reconstituted into DPhPC lipid bilayers (1 M NaCl, 15 mM Tris, pH 7.5, -50 mV). 1:80 protein:lipid ratio did not give nanopores in lipid bilayers. Errors are given as standard deviations, and were obtained from three different preparations of FraC nanopores.

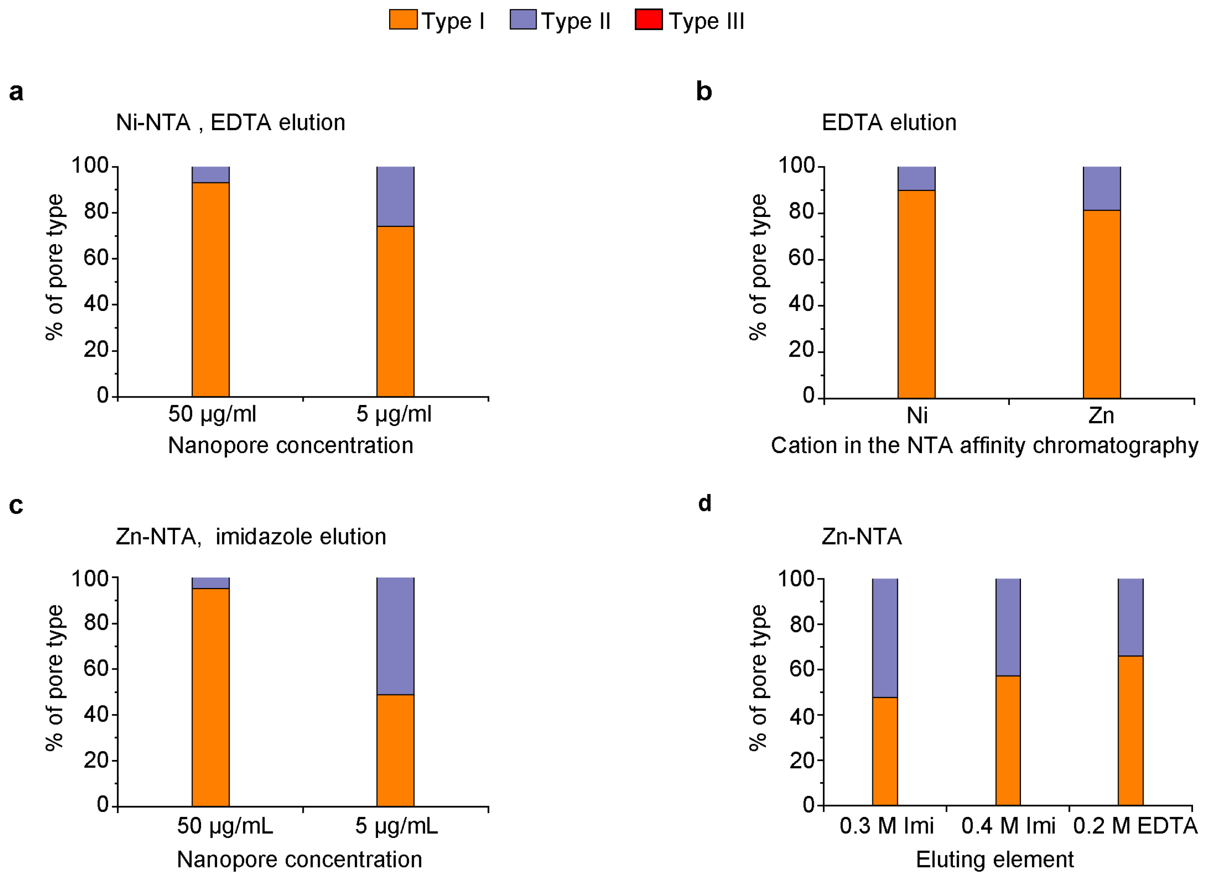

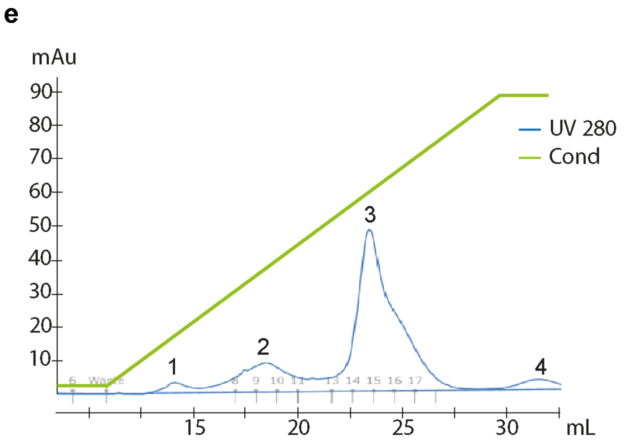

f

Peak	Count/percent of pores (pH 7.5)		
	Type I	Type II	Type III
1	NA	NA	NA
2	0	0	0
3	6/28.6%	15/71.4%	0
4	19/82.6%	4/17.4%	NA

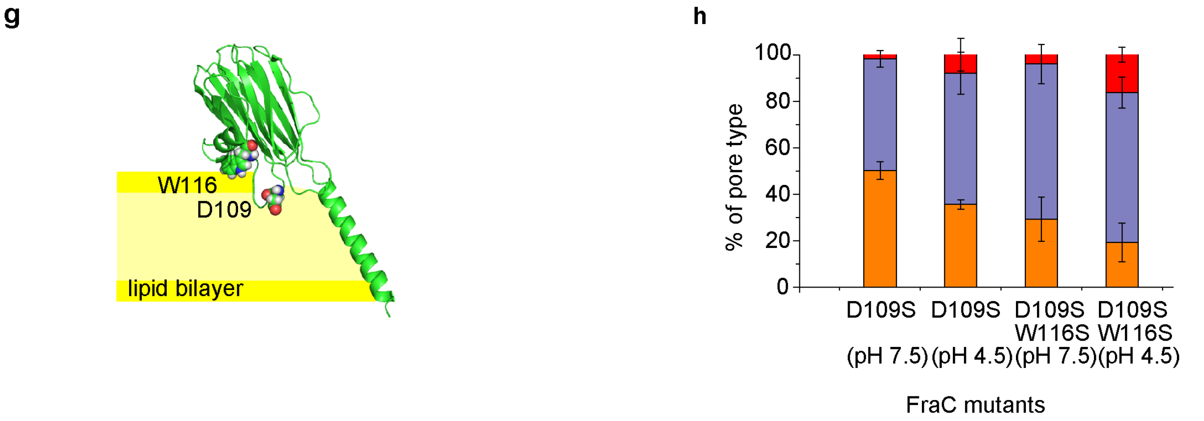

Supplementary Fig 2. Enrichment of type II Wt-FraC nanopores. **a** Effect of dilution on nanopore types. After FraC nanopores (0.5 mg/mL) were formed upon incubation with sphingomyelin / DPhPC liposome (1:10, protein:liposome ratio), the proteoliposome (100 µL) was solubilised by adding 10 µL of a 5% LDAO solution. The nanopores were then diluted to 50 µg/µL or 5 µg/µL by addition of buffer (SDEX: 150 mM NaCl, 15 mM Tris, pH 7.5) prior loading to a Ni-NTA column and eluted using 200 mM EDTA. High nanopore concentrations produced mainly type I Wt-FraC nanopores, while lower concentrations produced more of type II Wt-FraC nanopores. **b** Effect of the metal ion on the preparation of nanopore types. Nanopores were prepared as in (**a**) to a final concentration of 50 µg/µL and loaded to either a Ni-NTA or Zn-NTA chromatography column. Nanopores were then eluted using 200 mM EDTA. Lower affinity matrixes favoured the release of type II pores. **c** Same as (**a**), but using Zn-NTA affinity chromatography and eluting with 300 mM imidazole. **d** Effect of the strength of the elution buffer on the nanopore composition. Nanopores (5 µg/µL) prepared as in (**c**) were eluted with either 300 mM imidazole, 400 mM imidazole or 200 mM EDTA. The weaker the elution buffer, the higher amount of type II nanopores was found. **e** W112S-W116S-FraC oligomers were prepared and separated with FPLC chromatography using a HisTrap™ column as described in Methods. **f** Percentage of type I and type II nanopore from the purified fractions in (**e**). **g** Cartoon representation of one Wt-FraC protomer (PDB: 4TSY)[2]. One FraC protomer is shown as cartoon representation, while aspartic acid 109 and tryptophan 116 are shown as spheres. **h** pH dependence of the three nanopore types for D109S-FraC nanopores, showing that the mutation increased the proportion of smaller nanopores at pH 7.5. The nanopores were prepared as in (**a**) using a 5 µg/µL nanopore concentration prior loading

to Ni-NTA columns. Single channel recordings were performed under -50 mV applied potential in 1 M NaCl, 15 mM Tris, pH 7.5 except at pH 4.5, in which 1 M KCl, 0.1 M citric acid and 180 mM Tris base was used. Error bars stand for the standard deviations calculated from three repeats.

a

	pH 7.5				pH 4.5			
	Conductance (nS)	S.D.	n	d (nm)	Conductance (nS)	S.D.	n	d (nm)
**Wt-FraC**								
Type I	2.26	0.08	92	1.60	2.12	0.07	72	1.60
Type II	1.26	0.08	52	1.19	1.06	0.07	81	1.13
Type III	/	/	/	/	0.42	0.03	6	0.71
**W116S- FraC**								
Type I	2.14	0.09	101	1.60	2.07	0.1	57	1.60
Type II	1.24	0.06	53	1.22	1.08	0.09	92	1.13
Type III	/	/	/	/	0.41	0.03	53	0.71
**W112S-W116S-FraC**								
Type I	2.19	0.08	39	1.60	1.96	0.09	24	1.60
Type II	1.23	0.06	56	1.20	1.03	0.1	43	1.16
Type III	/	/	/	/	0.38	0.03	43	0.70
**D109S-FraC**								
Type I	2.22	0.11	48	1.60	2.09	0.09	50	1.60
Type II	1.25	0.07	48	1.20	1.04	0.07	78	1.13
Type III	0.41	0.04	2	0.69	0.41	0.02	12	0.71
**D109S-W116S- FraC**								
Type I	2.11	0.14	28	1.60	1.99	0.12	20	1.60
Type II	1.24	0.04	69	1.23	0.99	0.10	73	1.13
Type III	0.41	0.01	4	0.71	0.40	0.02	19	0.72

b

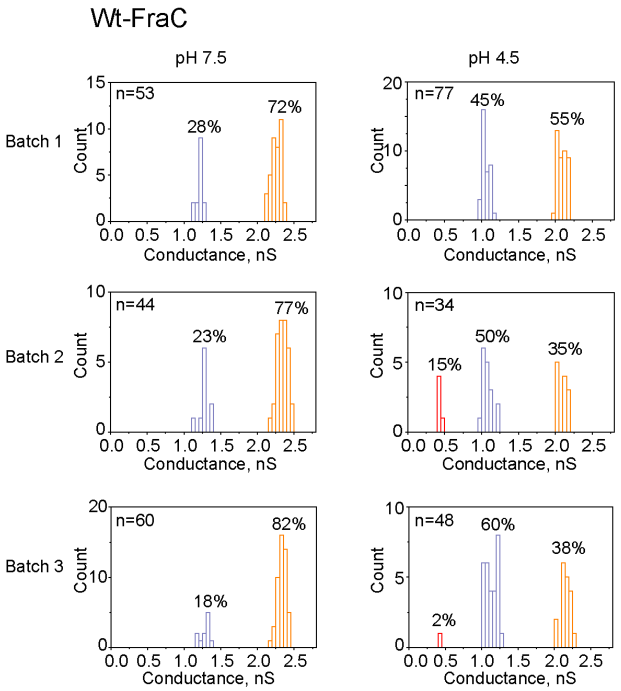

c

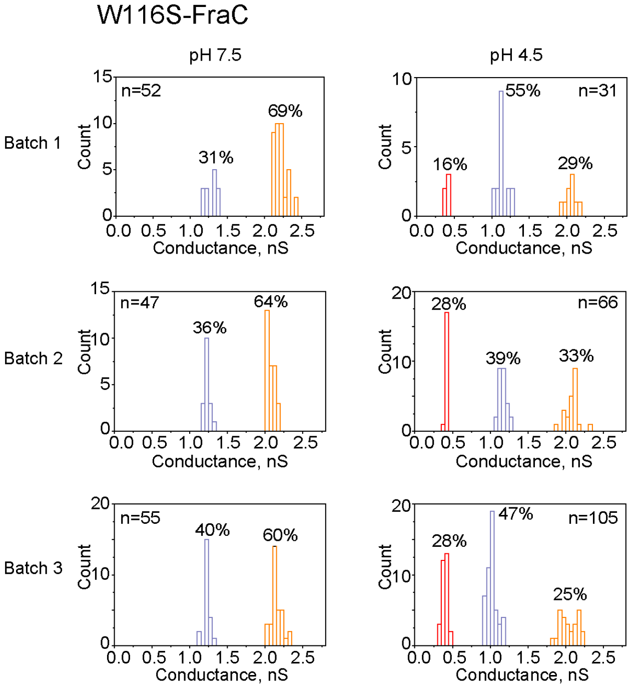

d

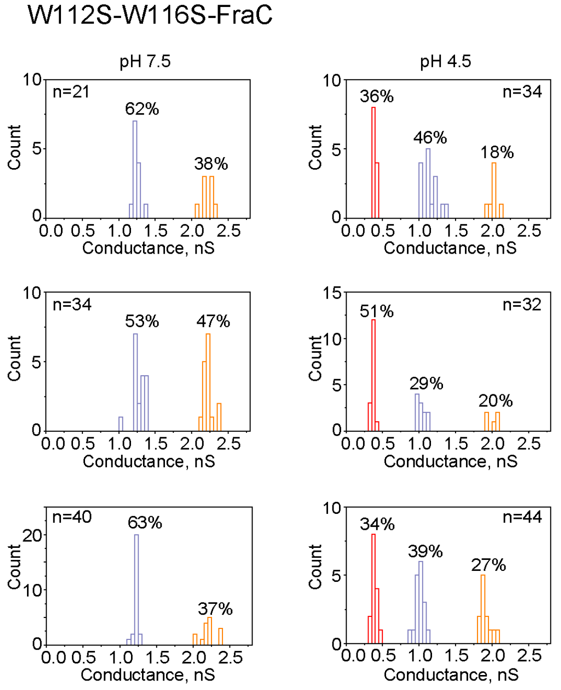

Fig.S3. continued

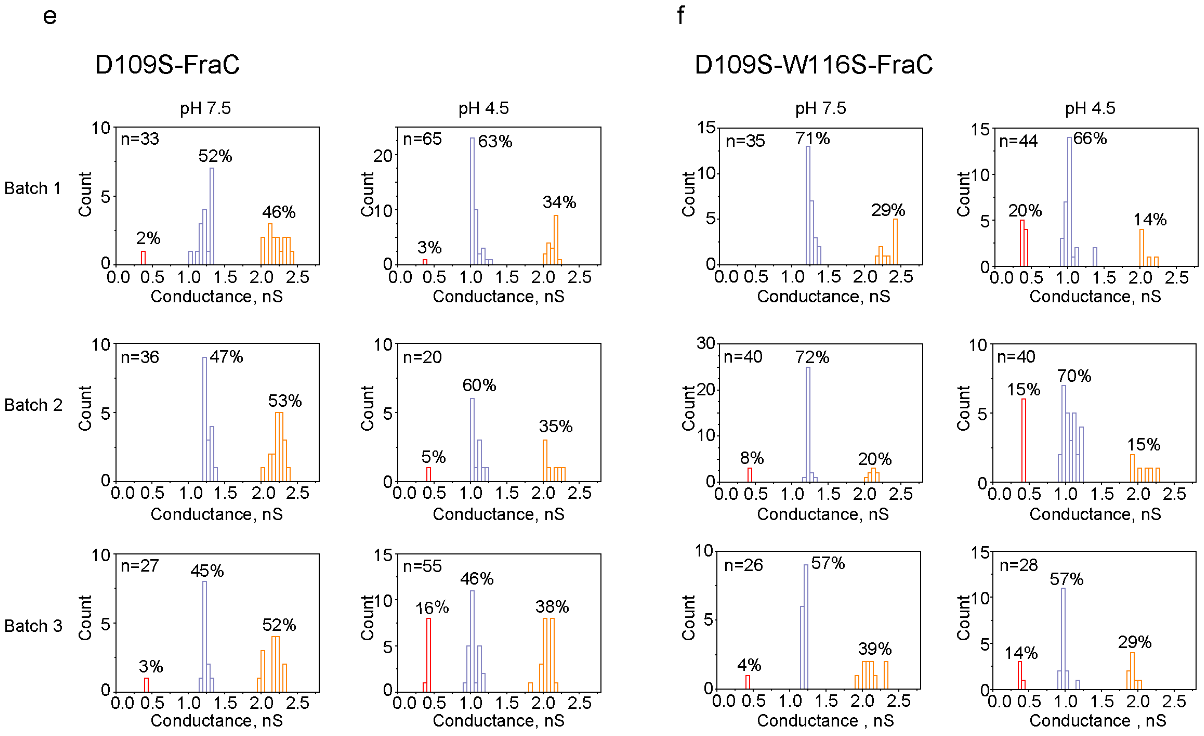

Supplementary Fig 3. Single channel conductance distributions of FraC nanopores at pH 7.5 and 4.5. **a** The table reports the average conductance values which were obtained by fitting Gaussian functions to conductance histograms. S.D. represents the standard deviation and 'n' is the number of individual nanopores tested. The diameter '*d*' of type I FraC was taken from the crystal structure (1.6 nm), while the diameters of type II and type III FraC nanopores were calculated from their conductance values using the formula: $\frac{d^2_{type\,I}}{d^2_{type\,x}} = \frac{G_{type\,I}}{G_{type\,x}}$, where $d_{type\,I}$ is the diameter of type I FraC, $d_{type\,x}$ is the diameter of type II or type III FraC, $G_{type\,I}$ is the conductance of type I FraC, $G_{type\,x}$ is the conductance of type II or type III FraC. It should be noticed that this formula is just an approximation, as it does not take into consideration the effect of the surface charge and electrical double layer

overlap inside the nanopore. **b-f** Each panel represents a different preparation of FraC nanopores as indicated. Single channels were collected under -50 mV applied potential. S.D. referred to standard deviations calculated from three repeats.

Supplementary Note 1

The different nanopore types most likely correspond to nanopores with different stoichiometry, rather than nanopores having the same stoichiometry but a different shape / geometry. Evidences supporting this interpretation are: **1)** Cryo-EM and crystal structure studies showed that FraC nanopores can adopt more than one oligomeric state[1,2]. **2)** The formation of the different nanopore types is concentration dependent (**Supplementary Figure 1, 2a**). **3)** The use of a chromatography matrix with lower affinity for his-tags (Zn-NTA versus Ni-NTA**, Supplementary Figure 2b**), the use of weaker eluting buffers (imidazole, versus EDTA, **Supplementary Figure 2c**) or elution with lower imidazole concentrations (**Supplementary Figure 2d**) favoured the formation of type II pores. The most likely interpretation to these results is that type II FraC has fewer subunits and fewer histidine tags than type I FraC nanopores, hence a lower affinity for the NTA matrix. **4)** W112S-W116S-FraC showed two bands in native-gels and one band was observed in denaturing SDS-gels (**Supplementary Figure 6**), suggesting the formation of nanopores with at least two different masses. **5)** The I-V curves of the three nanopore types showed similar rectification behaviours (**Supplementary Figure 4**), suggesting that the different nanopore types maintained a similar geometry.

We noticed that type II FraC nanopores inserted more efficiently at low pH. Therefore, to increase the production of type II nanopores at physiological pH, we exchanged aspartic acid at position 109, which is located at the lipid interface, for serine (**Supplementary Figure 2g-h**). As expected at pH 7.5 the fraction of type II nanopores increased from 23.0 ± 4.9% to 48 ± 3.6%, and a small fraction of type III nanopores appeared (**Supplementary Figure 3b,e**). The concomitant substitution of tryptophan at position 116 with serine

showed a further small increased in the fraction of type I and type II nanopores at pH 7.5 (**Supplementary Figure 3f**).

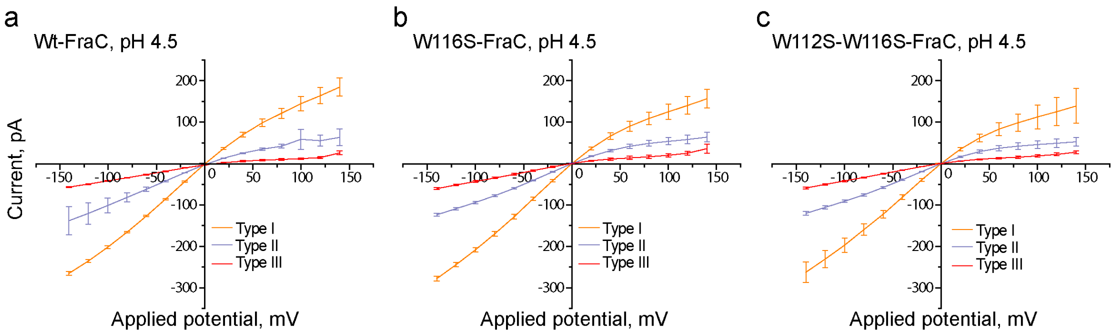

d

Voltage (mV)	Wt-FraC, Current(pA)±S.D.			W116S-FraC, Current(pA)±S.D.			W112-116S-FraC, Current(pA)±S.D.		
	Type I	Type II	Type III	Type I	Type II	Type III	Type I	Type II	Type III
-140	-261.3±4.8	-135.0±33.	-54.4±1.2	-278.0±5.1	-123.7±3.8	-60.8±2.3	-262.5±24.3	-120.3±4.5	-59.2±2.4
-120	-231.7±3.6	-117.5±25.	-47.0±1.3	-244.4±5.2	-109.0±2.9	-52.0±1.9	-230.8±20.4	-105.9±4.3	-50.5±1.8
-100	-198.6±3.2	-98.6±17.8	-39.4±1.2	-208.6±5.5	-94.2± 2.7	-43.5±2.0	-197.4±17.0	-91.3± 3.0	-42.2±1.5
-80	-162.8±1.8	-79.1±10.8	-32.1±1.0	-169.8±5.2	-77.9± 2.3	-35.0±1.8	-159.7±13.7	-75.7± 3.0	-33.7±1.2
-60	-123.9±1.6	-59.8±5.3	-24.3±1.0	-128.7±5.4	-60.1± 1.9	-26.2±1.9	-122.5± 9.1	-58.0± 2.3	-24.9±0.8
-40	-83.1±1.6	-39.6±1.7	-16.3±1.0	-85.1± 4.5	-40.7± 1.4	-17.0±1.8	-81.6± 5.9	-39.4± 1.7	-16.1±0.3
-20	-40.9±1.4	-19.7±0.2	-8.4±0.7	-41.6± 3.1	-20.4± 1.0	-8.0± 1.4	-39.8± 3.2	-19.5± 0.9	-7.6± 0.2
0	-0.1±0.2	-1.0±0.2	-1.3±0.1	-0.2± 0.5	-0.4± 0.3	0.2± 0.4	-0.2± 0.0	-0.3± 0.4	0.0± 0.4
20	38.1±2.2	15.0±0.5	4.3±0.7	36.3± 3.5	17.4± 1.2	6.5± 1.1	34.4± 4.0	16.2± 2.1	5.9± 0.5
40	71.3±5.6	27.3±1.3	8.0±1.4	66.3± 8.0	31.3± 3.2	10.5± 2.9	61.7± 9.2	28.8± 4.2	10.1± 1.2
60	99.8±9.5	36.7±2.6	10.3±1.7	89.8± 12.3	41.5± 5.4	13.5± 4.0	82.9± 15.1	36.8± 6.3	12.9± 2.0
80	122.9±12.0	43.7±4.4	11.9±2.0	108.8±15.4	48.4± 7.1	16.1± 4.4	98.2± 20.7	41.8± 7.7	15.2± 2.9
100	145.7±17.2	60.4±24.0	14.0±1.7	124.8±18.2	53.4± 8.6	19.7± 3.9	112.5± 28.8	45.7± 9.1	18.2± 3.2
120	164.9±19.5	57.0±13.2	16.4±1.9	140.1±21.3	58.0± 10.0	24.8± 4.8	125.2± 34.1	48.7± 10.3	21.9± 3.4
140	185.5±2	64.9±20.1	27.7±4.7	156.1±22.5	63.7± 11.4	35.7±10.9	138.8± 42.0	52.5± 10.8	27.4± 3.5

Supplementary Fig 4. Ionic current - voltage dependence for the three different types of FraC nanopores mutants. **a-c** I-V curves for Wt-FraC, W112S-FraC and W112S-W116S-FraC, respectively at pH 4.5. Error bars represent the standard deviations calculated from three repeats. **d** numerical values for the graphs in (**a-c**) at pH 4.5.

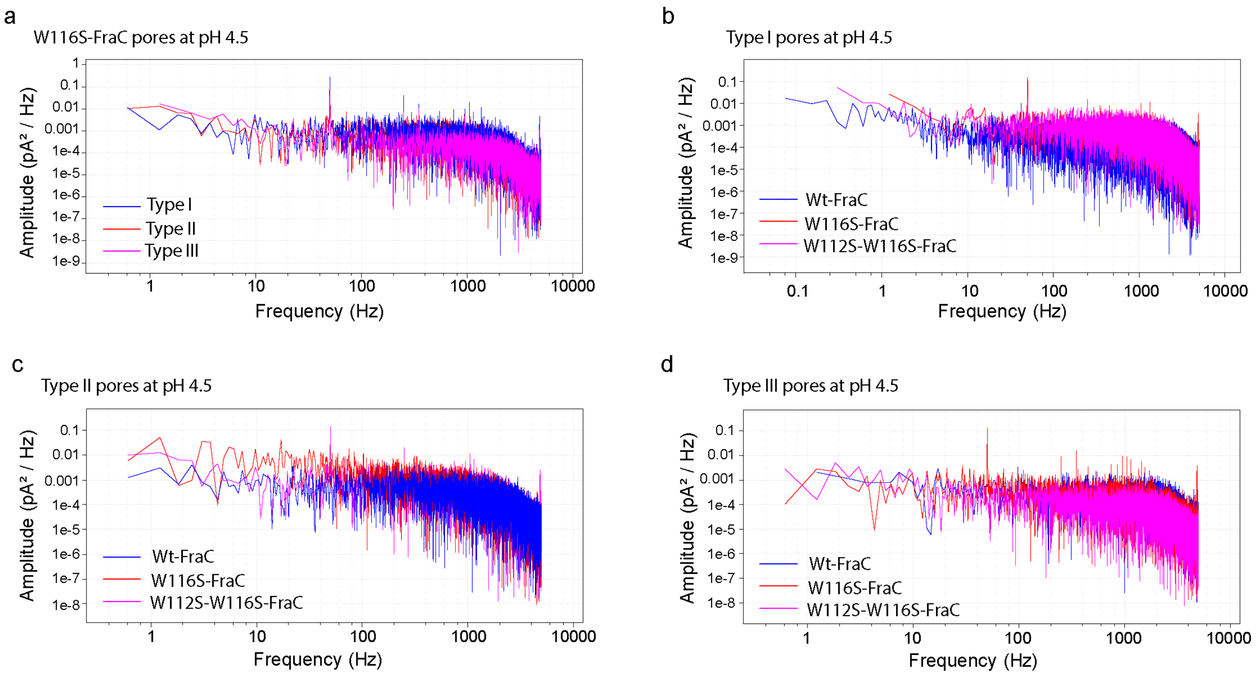

Supplementary Fig 5. Power spectrum of different types of pore at pH 4.5. **a** Power spectrum of different type pores of W116-FraC mutant. **b-d** Power spectrum of type I, type II, type III pores with Wt-FraC and mutants, respectively. The spectra were measured under -50 mV applied bias using a 10 kHz sampling rate and 2 kHz Bessel filter.

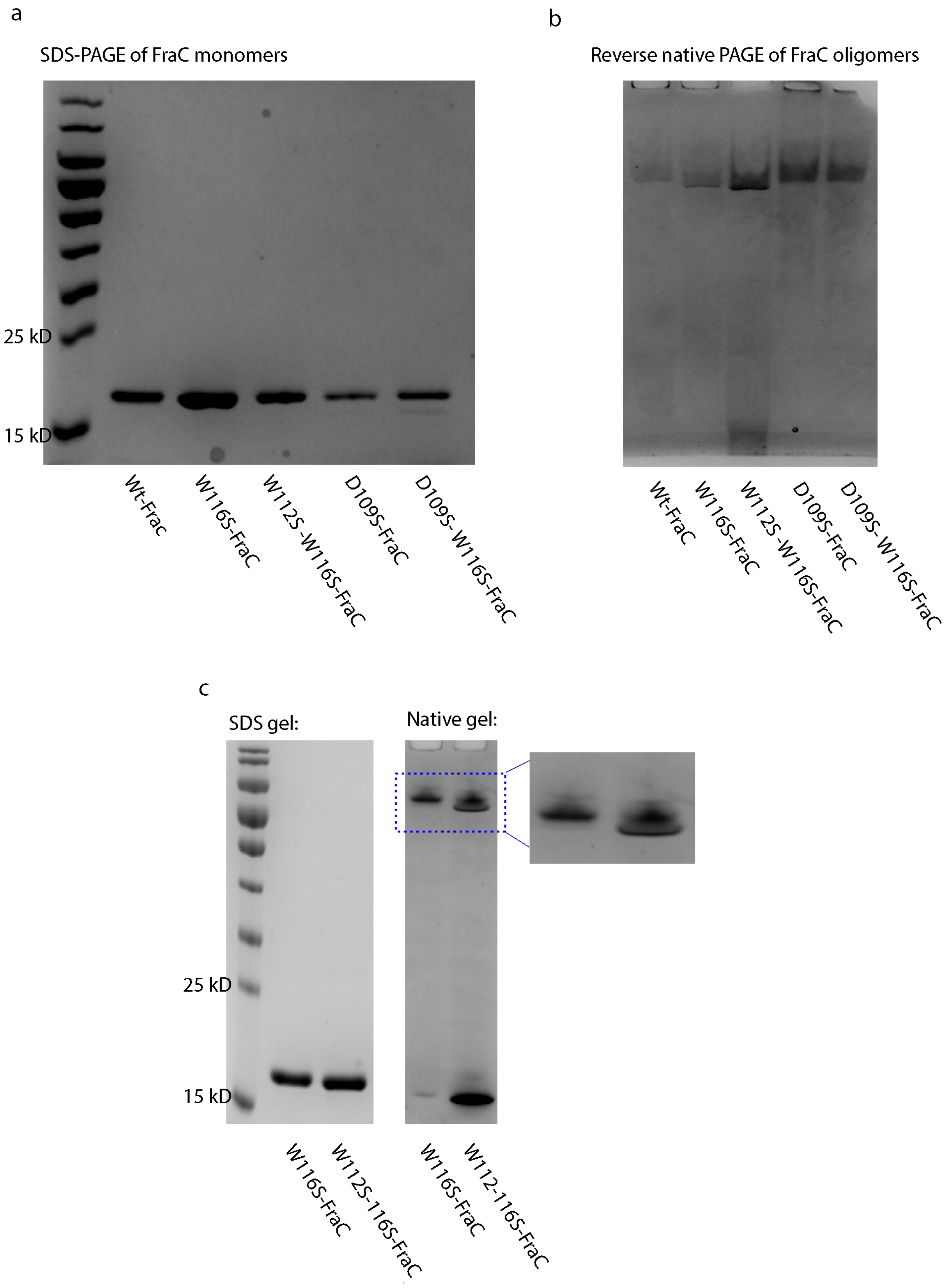
a
SDS-PAGE of FraC monomers
25 kD
15 kD
Wt-Frac
W116S-FraC
W112S-W116S-FraC
D109S-FraC
D109S-W116S-FraC
b
Reverse native PAGE of FraC oligomers
Wt-FraC
W116S-FraC
W112S-W116S-FraC
D109S-FraC
D109S-W116S-FraC
c
SDS gel:
Native gel:
25 kD
15 kD
W116S-FraC
W112S-116S-FraC
W116S-FraC
W112-116S-FraC

Supplementary Fig 6. Polyacrylamide gel electrophoresis (PAGE) of FraC monomers and oligomers. **a** 12% SDS-PAGE of FraC monomers. Wt-FraC is weakly expressed in *E. coli* cells and could not be observed in SDS-gels. Presumably this is because high concentrations of FraC might kill the host *E. coli* cells. Hence the pore-forming ability of Wt-FraC was neutralized by extending the N-terminus of FraC with a gene encoding for dihydrofolate reductase (DHFR). The two constructs were spaced by a furin digestion site [(DHFR)-GSSENRARYKRGSS-(FraC)-H_6]. DHFR-FraC monomers were then loaded to Ni-NTA affinity chromatography matrix and digested with trypsin (1 mg/mL). The released GSS-Wt-FraC (Wt-FraC in the figures) was then obtained and run in SDS-PAGE as a single band, along with all other FraC mutants. **b** 4-20% Tris-glycine Native PAGE of FraC nanopore oligomers. Because FraC has a net positive charge (+7.8 at pH 7.5), monomeric and oligomeric FraC nanopores do not migrate towards the cathode as in normal blue Native-PAGE. Thus, we switched the anode for the cathode, from which: "reverse native PAGE". Oligomers were loaded into the 4-20% Tris-glycine gels (Biorad) using 4X sample buffer (62.5 mM Tris, pH 6.8, 40% glycerol, 0.01% bromophenol blue) and a 300 V (reversed polarity) potential was applied for 90 minutes in a cold water bath. The running buffer was NOVEX NativePAGE^{TM} (Life technologies). Gels were then stained using coomassie dye (InstantBlue^{TM}, Expdedeon) for at least 1 hour. The samples in Tris buffer could give multiple bands. Interestingly, the FraC nanopores run at different heights in native gels bands, suggesting that nanopores have a different mass. **c** W112S-W116S-FraC and W116S-FraC nanopores showed multiple bands, which are likely to correspond to two different nanopore types as observed in electrical measurements.

Type II W116S-FraC

Angiotensinogen I:

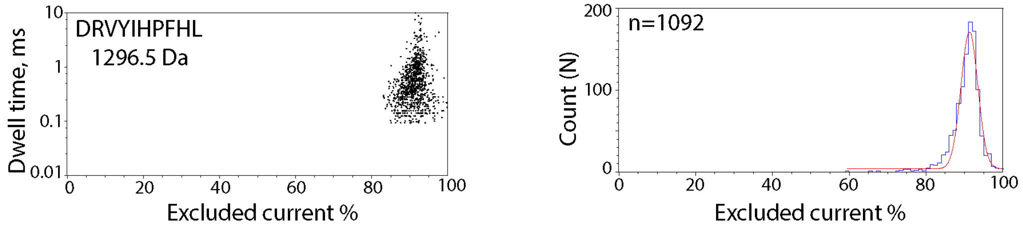

c-Myc 410-419

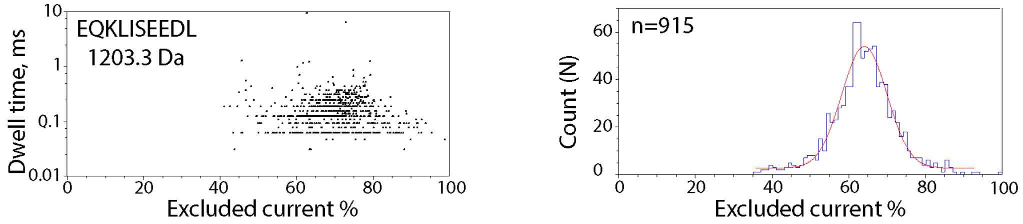

Angiotensin 1-9:

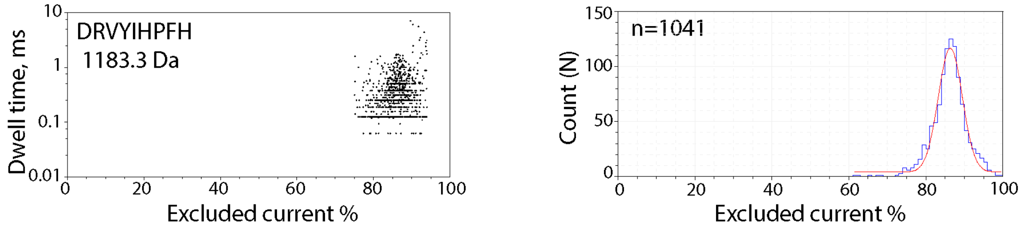

Neuropeptide-Like Protein 3 (NLP-3) (66-75):

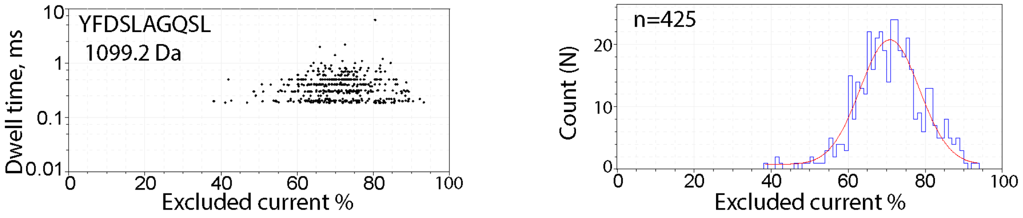

Angiotensin II:

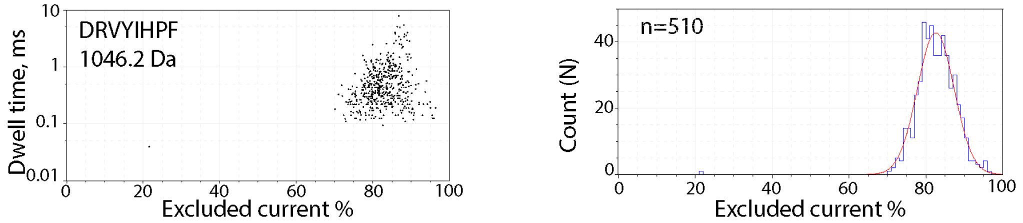

Asn1Val5 Angiotensin II:

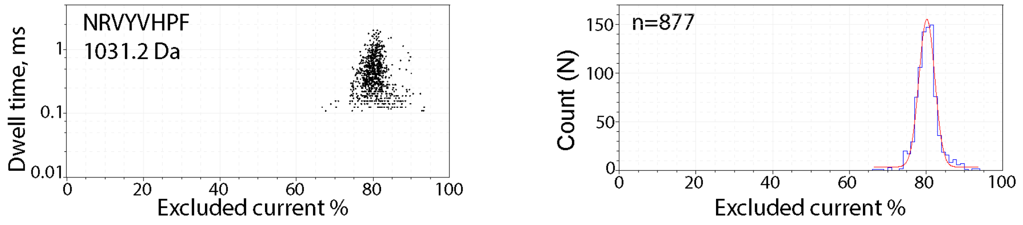

Supplementary Fig.6 continue

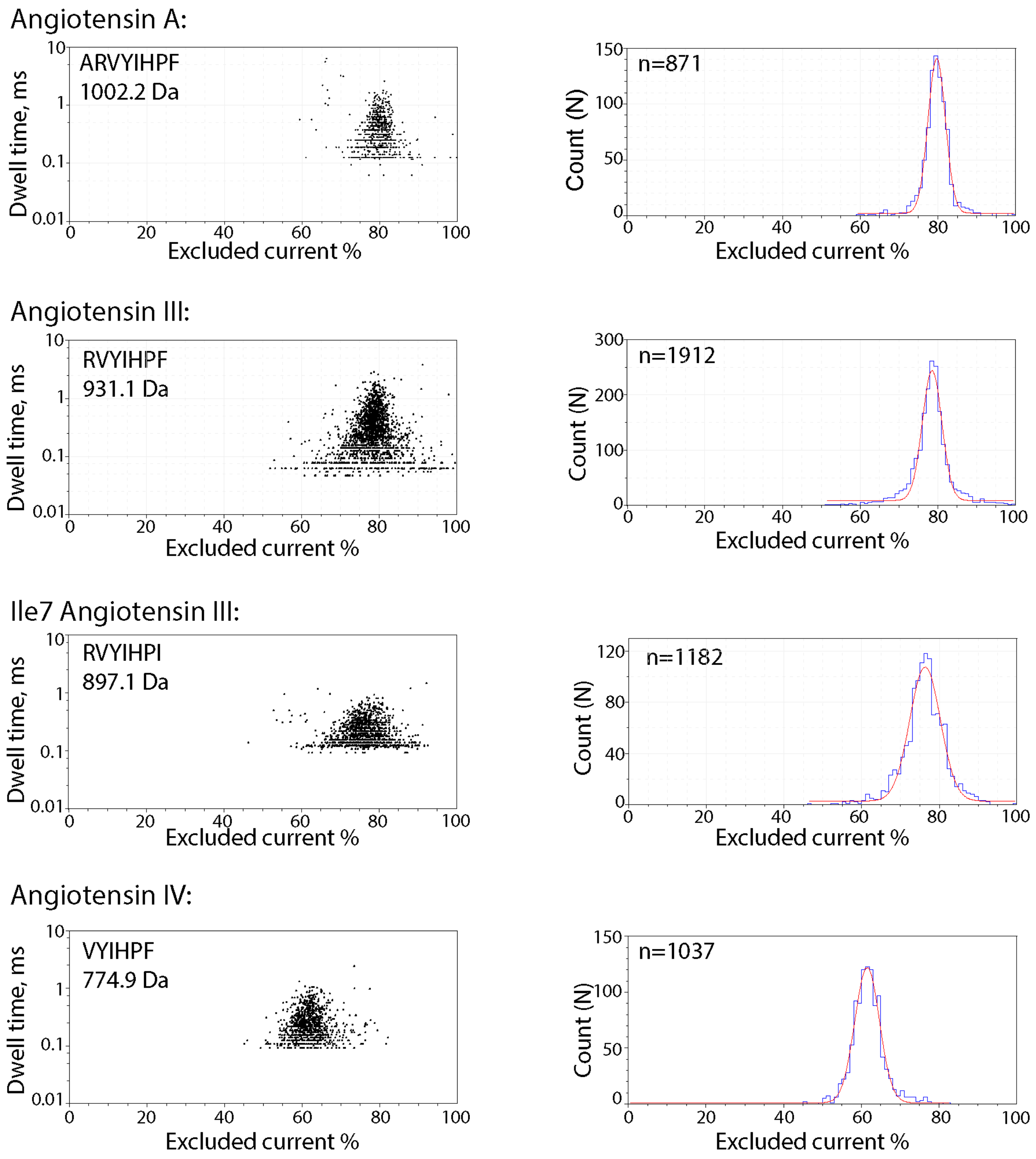

Supplementary Fig 7. Peptides characterization by type II W116S-FraC nanopores at pH 4.5. For each peptide indicated, on the left is shown a dwell time versus blockade $I_{ex\%}$ and on the right the respective event histogram for $I_{ex\%}$. The red line represents a Gaussian fit. Peptides were added to *cis* compartment and the recording were done using a 50 kHz sampling rate and a 10 kHz low pass filtering under -30 mV applied potentials.

a

Peptide sequence		M.W (g/mol)	Excluded current% (-30 mV)
Angiotensin III:	R V Y I H P **F**	931.09	77.9±0.5
Ile7-Angiotensin III:	R V Y I H P **I**	897.08	75.7±0.4

b c

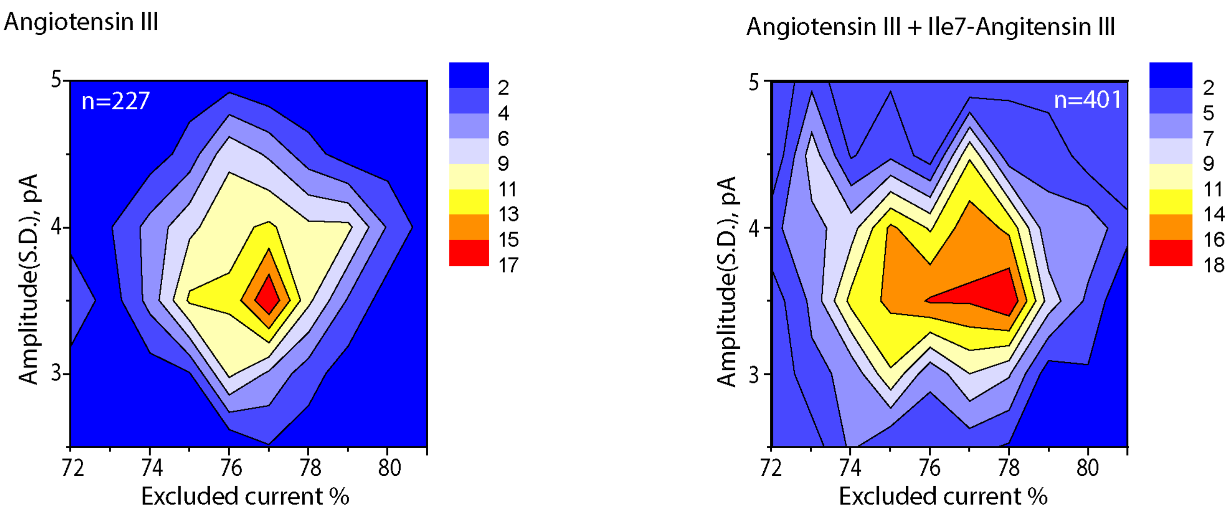

Supplementary Fig 8. Resolution of the FraC nanopore mass-spectrometer. **a** Sequence, molecular weight and $I_{ex\%}$ of angiotensin III and isoleucine-angiotensin III. **b, c** Color density plot of the $I_{ex\%}$ versus the amplitude standard deviations of current blockades when angiotensin III (**b**) and then isoleucine-angiotensin III (**c**) were added to the *cis* side of a type II W116S-FraC nanopore at pH 4.5. Standard deviations were calculated from minimum three repeats. Color density plot were created with Origin.

Supplementary Note 2

All peptides gave one main blockade with the exception of endomorphin I (**Supplementary Figure 10**), which induced two kinds of blockades to type III nanopores (**Supplementary Figure 11**). One blockade showed $I_{ex\%}$ = 99.1 ± 0.7 (level 1) and the other $I_{ex\%}$ = 80.3 ± 0.5 (level 2). In **Fig. 4c**, we used the $I_{ex\%}$ of level 2, which fitted well into the $I_{ex\%}$ versus mass curve with other peptides. The level 1 events showed a longer dwell time (4.0 ± 0.4 millisecond) compared to other peptide blockades (typically 0.5 millisecond or less). A likely explanation for the bimodal distribution of events is that level 1 events are due to peptide dimers blockades. Endomorphin-I (sequence: YPWF) contains several potential π-π interaction, and several studies have shown that aromatic peptides can form supramolecular assemblies[3]. Dilution of the analyte (10-fold, from 4 μM to 0.4 μM), did not change the relative amount of the level 1 blockades, which remained at 40% of the total blockades, suggesting that the association constant of the peptide dimers might be in the nM range.

a

Peptide sequence		M.W. (g/mol)	Excluded current% (-50 mV)
**Angio IV:**	V Y I H P F	774.4	98.9 ± 0.8
**Angio 4-8:**	Y I H P F	675.8	91.8 ± 0.4
**Endomorphin I:**	Y P W F	610.7	80.8 ± 0.5
**Leu-enkephalin:**	Y G G F L	555.6	65.5 ± 2.4

b

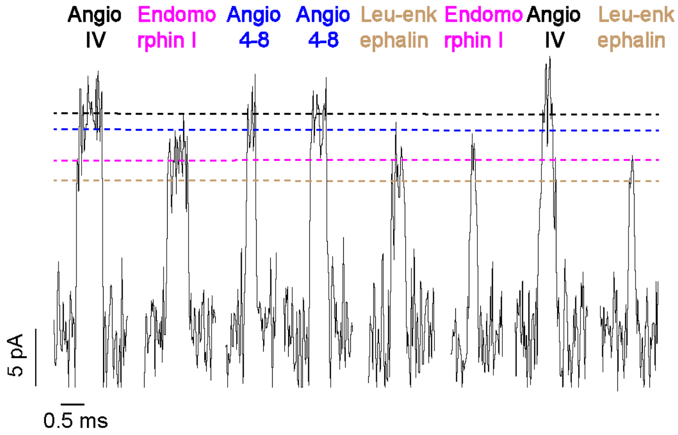

c

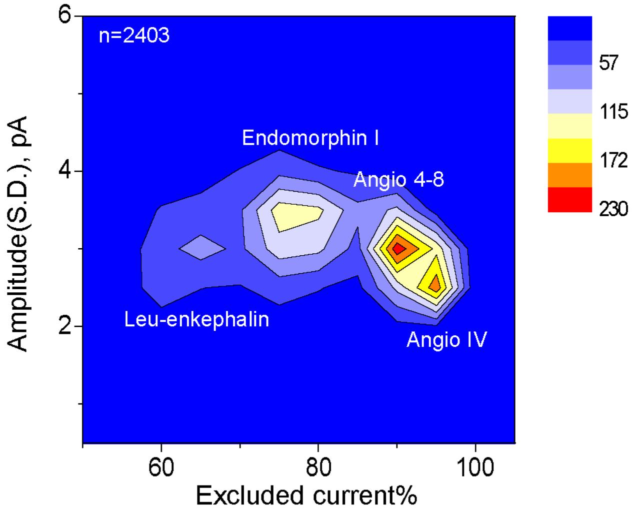

Supplementary Fig 9. Discrimination of short peptide mixture with type III W112S-W116S-FraC at pH 4.5. **a** Sequence, $I_{ex\%}$ (-50 mV) and M.W of angiotensin IV, angiotensin 4-8, endomorphin I and leucine enkephalin. **b** Selected blockades provoked by the different peptides. **c** Color density plot showing the $I_{ex\%}$ versus the standard deviation of the current blockade for the mixture of angiotensin IV, angiotensin 4-8,

endomorphin I and leucine-enkephalin. Standard deviations were calculated from minimum three repeats.

Type III W112S-W116S-FraC

Angiotensin IV:

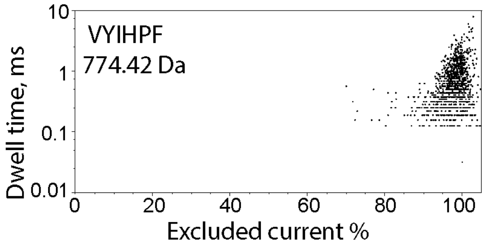

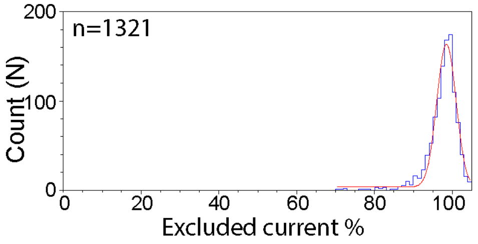

Angiotensin 4-8:

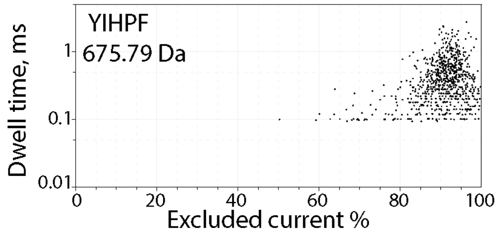

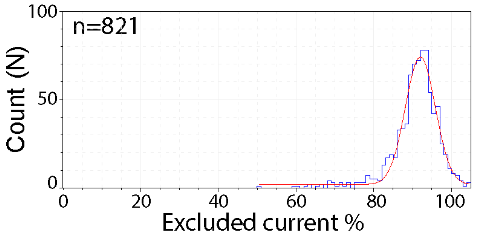

Endomorphin I:

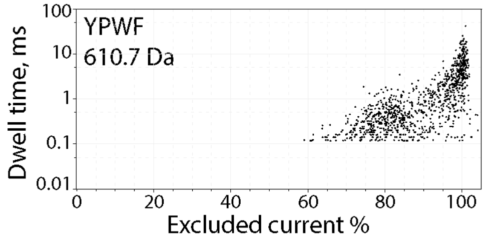

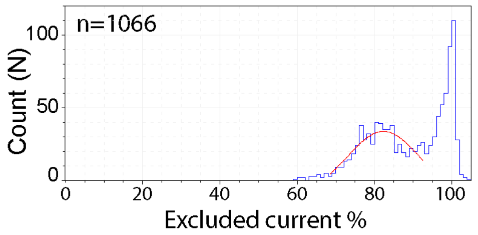

Methionine Enkephalin:

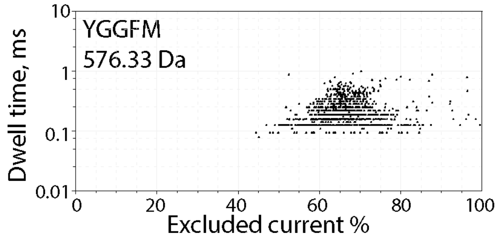

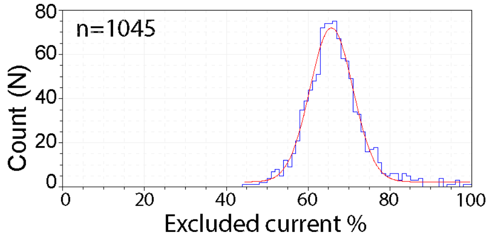

Leucine Enkephalin:

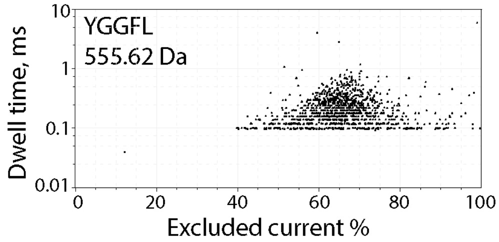

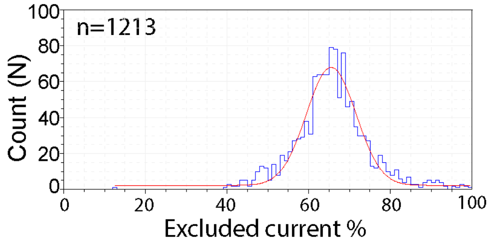

Supplementary Fig 10. Peptide characterization with type III W112S-W116S-FraC nanopores at pH 4.5. For each peptide indicated, on the left is shown a dwell time versus blockade $I_{ex\%}$ graph and on the right the respective event histogram for the blockade $I_{ex\%}$. The red line represents a Gaussian fit. Peptides were added to *cis* compartment and the recording were done using a 50 kHz sampling rate and a 10 kHz low pass filtering under -50 mV applied potentials.

a

Endomorphin I	Excluded current% (-50 mV)	Dwell time (ms)
Level 1	99.1 ± 0.7	3.99 ± 0.40
Level 2	80.3 ± 0.5	0.32 ± 0.04

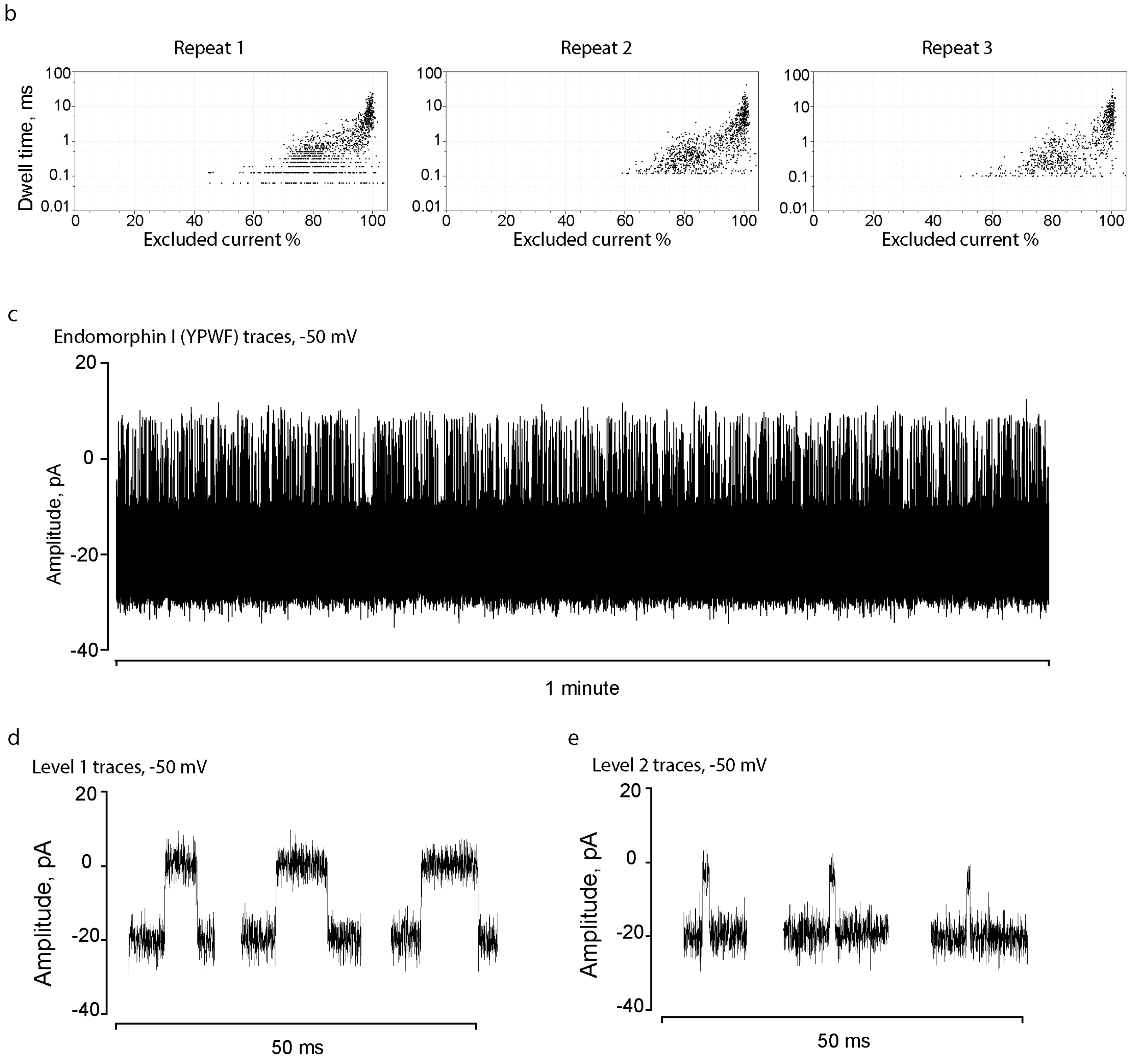

Supplementary Fig 11. Analysis of endomorphin I with type III W112S-W116S-FraC nanopore at pH 4.5 under -50 mV applied potential. **a** $I_{ex\%}$ versus dwell time of endomorphin I blockade. **b** Three repeats of $I_{ex\%}$ versus dwell time showing the two types of blockades. **c** Ionic current traces of endomorphin I recorded over one minute recording.

d,e Selected level 1 (**d**) and level 2 (**e**) events. Endomorphin I was added to the *cis* side of a type II W112S-W116S-FraC. Standard deviations were calculated from minimum three repeats.

Type I Wt-FraC

Endothelin 2:

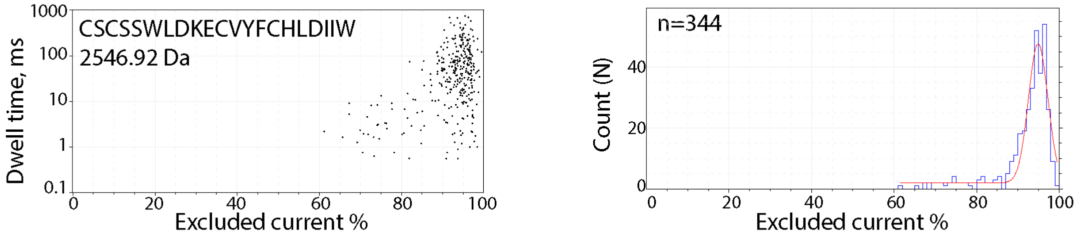

Endothelin 1:

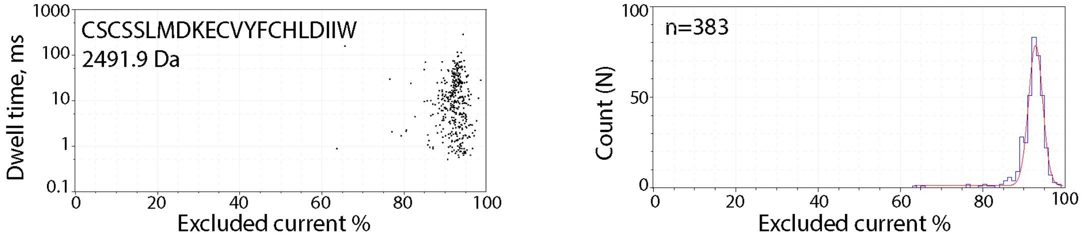

Dynorphin A porcine:

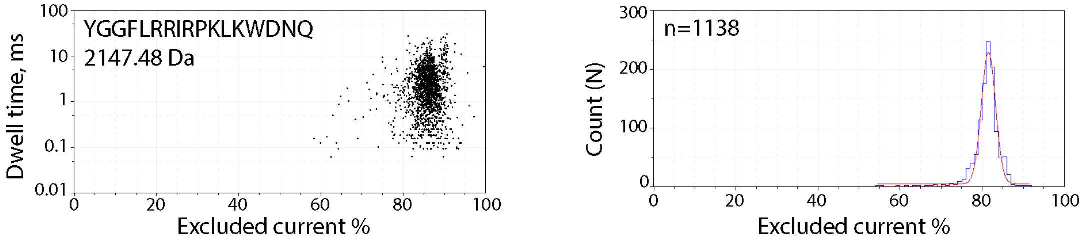

Pre-angiotensinogen:

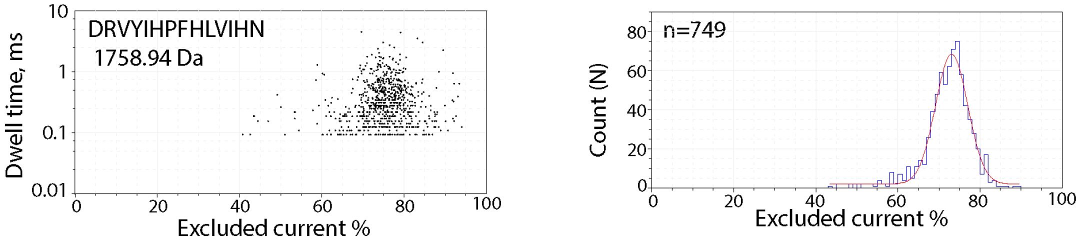

Angiotensin I (1296.48 Da)

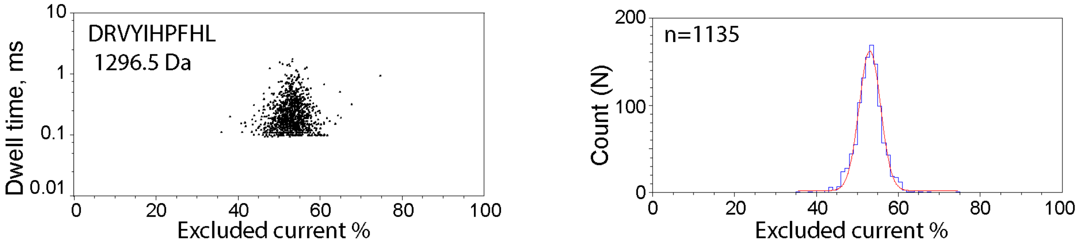

Supplementary Fig 12. Peptide characterization with type I Wt-FraC pore at pH 4.5. For each peptide indicated, on the left is shown a dwell time versus blockade $I_{ex\%}$ graph, on the right the respective event histogram for the $I_{ex\%}$. The red line represents a Gaussian fit. Peptides were added to *cis* compartment and the recording were done using a 50 kHz sampling rate and a 10 kHz low pass filtering under -30 mV applied potentials.

Supplementary Note 3

During peptide analysis, we noticed that, occasionally, *e.g.* when sampling angiotensin III at pH lower than four, small differences in the open pore currents of type II W112S-FraC nanopores gave relatively large difference in the $I_{ex\%}$ of the blocked peptide. This was not observed at pH 4.5. Therefore, when measuring the pH dependency of the $I_{ex\%}$ of the peptides (**Fig. 5**), we only considered type II W112S-FraC nanopores that under -30 mV applied potential showed an open current comprised between 27 and 30 pA at pH 4.5, between 25 and 26 pA at pH 4.0 and 3.8, and between 22 and 24 pA at pH 3.0.

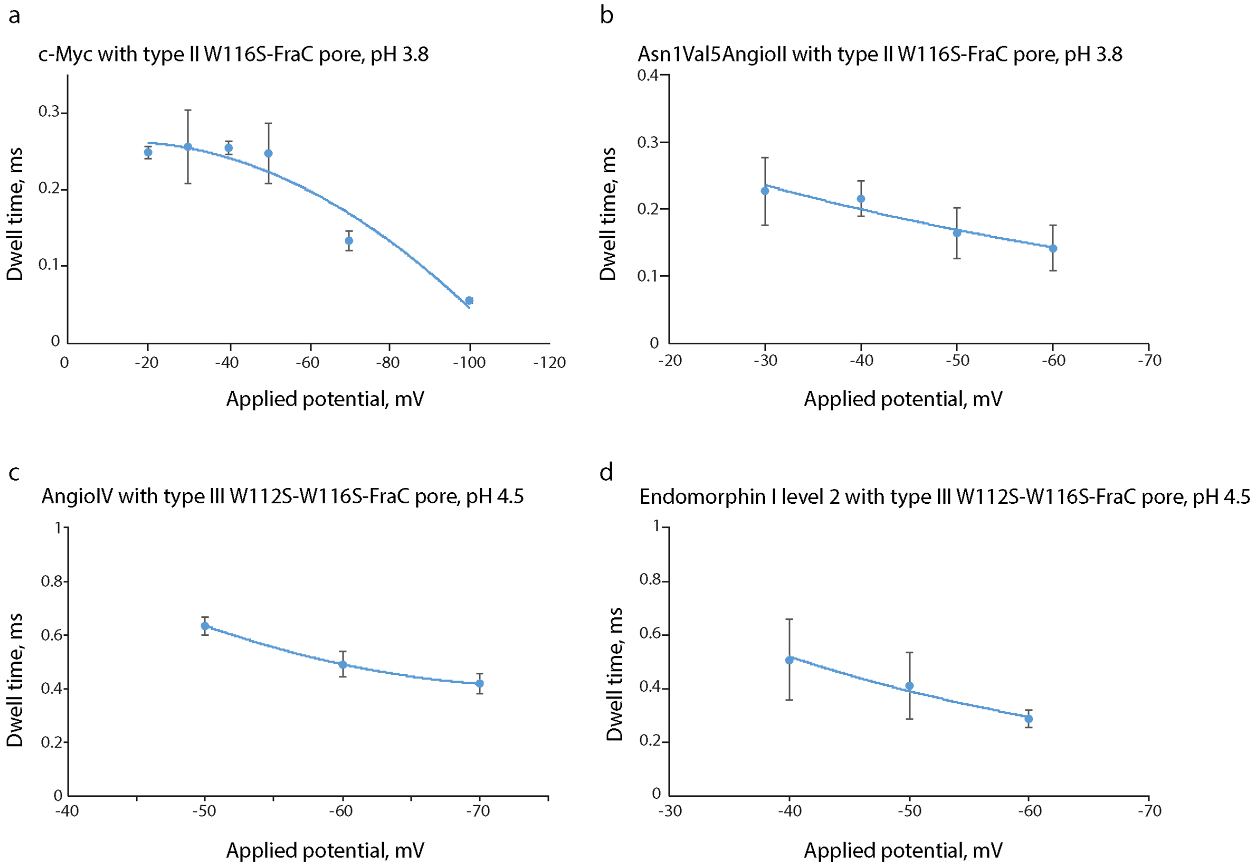

Supplementary Fig 13. Voltage dependence of the dwell time of four representative peptides using type II and type III FraC nanopores. **a,b** Voltage dependence of the dwell time of c-Myc and Asn1Val5 angiotensin II, measured with type II W116S-FraC at pH 3.8. **c,d** Voltage dependence of the dwell time of angiotensin IV and level 2 endomorphin I at pH 4.5, measured with type III W112S-W116S-FraC nanopore. Error bars represent the standard deviations calculated from three repeats

Supplementary Table 1. Ion selectivity of different FraC nanopores at pH 7.5 and 4.5**.** The ion selectivity (P_{K^+}/P_{Cl^-}) was calculated from the reversal potential according to the Goldman-Hodgkin-Katz equation[4]: $\frac{P_{K^+}}{P_{Cl^-}} = \frac{[a_{Cl^-}]_{trans} - [a_{Cl^-}]_{cis} e^{V_r F/RT}}{[a_{K^+}]_{trans} e^{V_r F/RT} - [a_{K^+}]_{cis}}$, where *Vr* is the reversal potential, P_{K^+}/P_{Cl^-} the ion selectivity, *a* the activity of ions and *F* the Faraday constant. Electrical recordings were carried out with 1960 mM KCl in the *cis* solution and 467 mM KCl in the *trans* solution. The activity of ions was calculated by multiplying the molar concentration of the ion for the mean ion activity coefficients (0.649 for 500 mM KCl, and 0.573 for 2000 mM)[5]. Standard deviations were calculated from minimum three repeats.

		pH 7.5		pH 4.5	
		Reversal potential (mV)	P_{K^+} / P_{Cl^-}	Reversal potential (mV)	P_{K^+} / P_{Cl^-}
Wt-FraC	Type I	17.2±1.2	3.6±0.4	10.5±1.4	2.1±0.2
	Type II	20.8±1.6	5.2±0.9	12.3±1.2	2.4±0.2
	Type III	/	/	20.6±1.1	5.0±0.6
W116S -FraC	Type I	/	/	10.1±0.9	2.0±0.1
	Type II	/	/	12.8±0.7	2.5±0.2
	Type III	/	/	18.8±0.5	4.2±0.2
W112S-W16S-FraC	Type I	/	/	8.8±1.2	1.9±0.2
	Type II	/	/	14.0±0.1	2.8±0.1
	Type III	/	/	20.1±0.6	4.8±0.3

Supplementary References

1. Mechaly, A. E. *et al.* Structural insights into the oligomerization and architecture of eukaryotic membrane pore-forming toxins. *Structure* **19,** 181–191 (2011).
2. Tanaka, K., Caaveiro, J. M. M., Morante, K., González-Manãs, J. M. & Tsumoto, K. Structural basis for self-assembly of a cytolytic pore lined by protein and lipid. *Nat. Commun.* **6,** 4–6 (2015).
3. Wei, G., Gazit, E. & Aviv, T. Structural Polymorphism in a Self-Assembled Tri-Aromatic Peptide System. *ACS Nano* **12,** 3253–3262 (2018).
4. Gu, L.-Q. *et al.* Reversal of charge selectivity in transmembrane protein pores by using noncovalent molecular adapters. *Proc. Natl. Acad. Sci.* **97,** 3959–3964 (2000).
5. Lide, D. R. CRC Handbook of Chemistry and Physics, 84th Edition, 2003-2004. *Handb. Chem. Phys.* **53,** 2616 (2003).
